# TargetClone: A multi-sample approach for reconstructing subclonal evolution of tumors

**DOI:** 10.1371/journal.pone.0208002

**Published:** 2018-11-29

**Authors:** Marleen M. Nieboer, Lambert C. J. Dorssers, Roy Straver, Leendert H. J. Looijenga, Jeroen de Ridder

**Affiliations:** 1 Center for Molecular Medicine, University Medical Center Utrecht, Utrecht, The Netherlands; 2 Department of Pathology, Erasmus MC Cancer Institute, University Medical Center Rotterdam, Rotterdam, The Netherlands; 3 Princess Maxima Center for Pediatric Oncology, Utrecht, The Netherlands; Roswell Park Cancer Institute, UNITED STATES

## Abstract

Most tumors are composed of a heterogeneous population of subclones. A more detailed insight into the subclonal evolution of these tumors can be helpful to study progression and treatment response. Problematically, tumor samples are typically very heterogeneous, making deconvolving individual tumor subclones a major challenge. To overcome this limitation, reducing heterogeneity, such as by means of microdissections, coupled with targeted sequencing, is a viable approach. However, computational methods that enable reconstruction of the evolutionary relationships require unbiased read depth measurements, which are commonly challenging to obtain in this setting. We introduce TargetClone, a novel method to reconstruct the subclonal evolution tree of tumors from single-nucleotide polymorphism allele frequency and somatic single-nucleotide variant measurements. Furthermore, our method infers copy numbers, alleles and the fraction of the tumor component in each sample. TargetClone was specifically designed for targeted sequencing data obtained from microdissected samples. We demonstrate that our method obtains low error rates on simulated data. Additionally, we show that our method is able to reconstruct expected trees in a testicular germ cell cancer and ovarian cancer dataset. The TargetClone package including tree visualization is written in Python and is publicly available at https://github.com/UMCUGenetics/targetclone.

## Introduction

Tumors develop from the accumulation of somatic mutations over time. In a tumor, often various subclonal populations with (partially) overlapping mutation patterns co-exist. These subclones are formed through an evolutionary process [1–3]. Reconstructing the subclonal evolution is important, as it can assist in characterizing the mutations driving tumor development and progression, and can be helpful to decipher the mechanisms underlying treatment response [4, 5].

A number of algorithms have been developed to reconstruct subclonal evolution trees from rapidly emerging next-generation sequencing data (S1 Fig). The existing methods can coarsely be divided into two categories, those based on somatic single-nucleotide variants (SNVs) and those based on somatic copy number variations (CNVs). Somatic SNV-based methods, such as LICHeE, PhyloSub, TrAp and AncesTree, are most often based on two important assumptions; the sum-rule assumption and infinite sites assumption (ISA) [6–9]. Based on the sum rule, a branched tree, rather than a linear tree, can be ruled out if the sum of the variant allele frequency (VAF) of SNVs in the child subclones is larger than the VAF of SNVs in the parent [7]. Under the ISA, somatic SNVs are not expected to be gained twice independently. Furthermore, somatic SNVs are not expected to be lost once gained. An important limitation is that the VAF is affected by CNVs. As a result, SNV-based methods are restricted to using somatic SNVs in copy number-neutral regions. To overcome potential loss of information due to these restrictions, alternative methods, such as CNTMD, ThetA, TITAN, MEDICC, CloneCNA and CLImAT-HET, have been developed that aim to either infer the copy numbers of subclones, or reconstruct (subclonal) evolution trees from CNVs inferred from e.g. read depth information [10–15]. Additionally, the PhyloWGS algorithm combines somatic SNVs and CNVs to further increase the tree reconstruction accuracy [16]. However, using read depth to determine the copy number of individual subclones in heterogeneous tumor populations is a challenging problem, as such populations consist of several subclones and non-tumor cells mixed in different unknown fractions [3, 15, 17]. It is therefore hard to distinguish between CNVs and differences in subclonal fraction, and multiple combinations of subclonal fraction and subclonal CNVs may explain the overall read depth profile.

While single-cell sequencing approaches largely mitigate the problem of sample heterogeneity, it is currently not yet possible to sample accurate representations of the entire subclonal diversity using these techniques [18–20]. Therefore, an interesting alternative is to perform microdissections to obtain multiple samples of the same tumor (S2 Fig), while at the same time reducing sample heterogeneity [21–23]. However, the typical low read depth of whole genome sequencing (WGS) data complicates the inference of somatic SNVs and CNVs in any sample, and in microdissections in particular [16, 24, 25]. Targeted sequencing-based approaches, including whole exome sequencing (WES), have resulted in a higher coverage, but lead to variable and biased read depth across the genome that may limit accurate detection of CNVs [17, 26–31]. Currently, no methods exist that can be used to unravel subclonality directly from the uncorrected read depth data measured with targeted sequencing. Here, we present TargetClone, a method to reconstruct subclonal evolution of tumors from only SNP allele frequencies and somatic SNVs, which does not rely on read depth or CNVs and thus does not require additional corrections. TargetClone is geared towards inferring trees from targeted sequencing data from microdissected samples.

**Fig 1:**
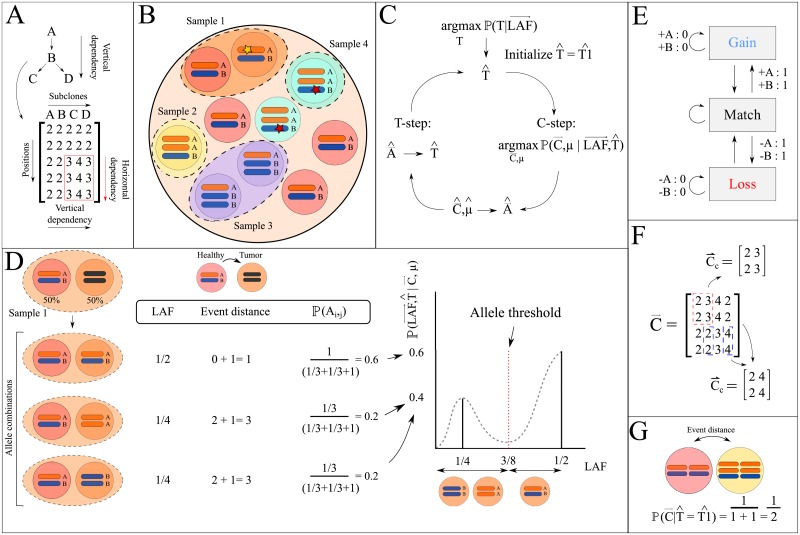
Overview of the TargetClone methodology. (A) A vertical dependency exists between subclones A-D. In red a horizontally dependent region is highlighted. (B) Multiple subclones with different somatic mutation patterns are sampled from a tumor. Sample 1 contains a mixture of tumor cells and healthy cells, while sample 2, 3 and 4 only contain tumor cells. A star indicates a somatic SNV. (C) General overview of the iterative optimization used in TargetClone. (D) A sample containing healthy cells and a tumor component (each present in 50% of the sample) with a copy number of 2 can be explained by 3 possible scenarios (see left) that each result in a different LAF measurement. Each scenario is scored using the event distance to generate a probability distribution (right). The alleles of the tumor component can be derived from the probability distribution. (E) The FST used to compute the event distance between subclones. Every allele can be gained or lost, which is assigned a distance of 1. If the adjacent position is affected by the same event, the distance is not increased further, which is indicated by the loops to the same state. (F) Two $\vec{\left(C_{c}\right)}$ with a different combination of parent and child subclone are highlighted with the blue and red dashed lines. (G) Computation of $P(\vec{C}|\hat{T})$ for two adjacent alleles. Using the horizontal dependency, the event distance equals 1.

TargetClone is mainly based on three assumptions. First, it assumes that the input samples contain one major tumor subclone, which have for example been acquired through microdissection as was discussed in the previous paragraph. Contamination with other subclones is allowed, as long as one subclone is dominant in the sample. Second, due to the existence of evolutionary relationships between all subclones in a tumor sample, the subclones are expected to exhibit (partial) overlap in their mutation patterns [6, 9, 32]. In combination with the assumption that somatic mutations accumulate over time and are not lost, we assume that subclones with major overlapping mutation patterns are more closely related than subclones with very distinct mutation patterns (vertical dependency) (Fig 1A) [7, 8]. Thus, we can add direction to the subclonal evolution trees, as the parent of a subclone should have a smaller set of mutations. Third, as our method aims to reconstruct evolutionary trees, we integrate the horizontal dependency assumption to more accurately estimate evolutionary distances between subclones as was previously described in MEDICC [13]. The horizontal dependency works by assuming that two adjacent measurements on the genome are likely dependent, and thus have a high probability of being affected by the same CNV event (Fig 1A).

We demonstrate the performance of our method on simulated data and two real data cases. The first real dataset consists of four type II non-seminomatous (NS) Testicular Germ Cell Cancers (TGCC) with intrinsic resistance to chemotherapy [33]. For each tumor, multiple histological elements have been macro- and microdissected. Allele frequencies (AF) and somatic SNVs were measured with targeted sequencing [23]. Second, we aimed to demonstrate that TargetClone can also be applied to another tumor type. Thus, we ran our method on a dataset consisting of multiple primary tumor and metastasis samples with reduced heterogeneity of an ovarian cancer patient [34]. In this dataset, the AF were measured using a SNP array, and somatic SNVs were measured using targeted sequencing.

## Materials and methods

### Definitions

The method accepts *m* purified samples of the tumor bulk, which can be obtained through e.g. microdissection. As a result of the reduced heterogeneity, we make the assumption that samples consist of one major tumor subclone and are potentially mixed with healthy cells (Fig 1B), although we later show that TargetClone is robust to moderate levels of contamination from other subclones. The fraction of the major tumor subclone in the sample is denoted as the scalar *μ*, and hence, the fraction of healthy cells in the sample can be computed as 1—*μ*. Each sample can have a different *μ*.

We assume that the AF have been measured at *n* heterozygous Single-Nucleotide Polymorphism (SNP) positions in the matched healthy genome that are informative for detecting allelic imbalance. In this text, the term AF measurements will refer to the fraction of the non-reference allele measured at these SNP positions. Furthermore, we assume that in every sample the AF of somatic SNVs have been measured, which will be referred to as somatic SNV measurements. The AF measurements of the SNPs and the AF measurements of the somatic SNVs are used as input to TargetClone.

The AF are represented in a matrix $\vec{AF}=\left[AF_{i,j}\right]$, where *AF*_*i*,*j*_ represents the measured AF at SNP position *i* in subclone *j*. From the AF measurements, lesser allele frequency (LAF) measurements are computed as 1—*AF*_*i*,*j*_ for every *AF*_*i*,*j*_ larger than 0.5. The LAF measurements are represented in matrix $\vec{LAF}$, which is in the same format as matrix $\vec{AF}$.

The copy numbers of the subclones can be represented in matrix $\vec{C}=\left[C_{i,j}\right]$, where $C_{i,j}\in N$ represents the copy number of subclone *j* at AF measurement position *i*. Consistent with the assumption that every sample may contain healthy cell admixture, the first column of $\vec{C}$ will always contain the copy numbers of healthy cells, which are assumed to be 2 (Fig 1A). Similar to the copy numbers, the alleles of the *m* samples can be represented in a matrix $\vec{A}=\left[A_{i,j}\right]$. *A*_*i*,*j*_ denotes the alleles that are present at this AF measurement position, which will be referred to as allele A (reference) or B (variant). For example, *A*_*i*,*j*_ could be AB or ABB. The total number of alleles equals the copy number at each position. The first column of $\vec{A}$ also represents the alleles of healthy cells, which are assumed to be AB. The rows in $\vec{C}$ and $\vec{A}$ are ordered by AF measurement position on the genome. The ordering of the columns (with the exception of the first column) is arbitrary.

TargetClone outputs estimates of the copy numbers ($\vec{C}$) and alleles ($\vec{A}$), the tumor fraction (*μ*) per sample, and an estimate of the subclonal evolution tree (*T*), which describes the relations between the input samples and an estimated distance between these.

### Model

The objective of TargetClone is to infer the subclonal evolution tree *T* from the AF and somatic SNV measurements (Fig 1C):

$$\begin{array}{c} \underset{T}{arg max}P\left(T|\vec{AF},\vec{SNV}\right) \end{array}$$ (1)

Eq 1 is optimized using an iterative heuristic model, consisting of a **T-** and **C-step**:

**T-step**: a tree $\hat{T}$ is inferred from $\hat{\vec{A}}$, the AF measurements, and somatic SNV measurements. $\hat{\vec{A}}$ can be estimated from $\hat{\vec{C}}$ and $\hat{\mu }$, which are both inferred by the model in the **C-step**.

**C-step**: we maximize the likelihood of observing $\vec{C}$ and *μ* given the LAF measurements per sample, which are derived from the AF measurements, and the current estimate of the subclonal evolution tree $\hat{T}$:

$$\begin{array}{c} \underset{\vec{C},\mu }{arg max}P\left(\vec{C},\mu |\vec{LAF},\hat{T}\right) \end{array}$$ (2)

The model is initiated with an estimate of the subclonal evolution tree, $\hat{T1}$. By default, we assume that all subclones have a healthy cell as the last known common precursor. Thus, in our initial tree the healthy cell is set as the parent of every tumor subclone. However, starting the model from a different precursor with allele compositions other than AB is also possible.

We demonstrate that starting the model with different initial trees does not affect the results, showing that the method is robust for different starting points.

The **T** and **C steps** are repeated iteratively until $\hat{T}$ has converged. The tree is considered converged when the edges and the total distance between all subclones equals that of a tree that has been reconstructed in any previous iteration.

### C-step

Eq 2 can be rewritten as the following using Bayes’ rule (see S1 Text for the full derivation):

$$\begin{array}{c} P\left(\vec{C},\mu |\vec{LAF},\hat{T}\right)\propto P\left(\vec{LAF}|\vec{C},\mu ,\hat{T}\right)P\left(\vec{C}|\;\hat{T}\right) \end{array}$$ (3)

The computation of $P(\vec{LAF}|\vec{C},\mu ,\hat{T})$ and $P(\vec{C}|\;\hat{T})$ are explained below.

#### Computing $P(\vec{LAF}|\vec{C},\mu ,\hat{T})$

For a single measurement position *i* and some subclone *j*, $P(LAF_{i,j}|C_{i,j},\mu ,\hat{T})$ is computed by enumerating all possible alleles that can result from the copy number *C*_*i*,*j*_, which can easily be performed for realistic *C*_*i*,*j*_. For example, if *C*_*i*,*j*_ = 2, the tumor subclone can contain the alleles AA, BB or AB (see Fig 1D), which we will denote as the set *Q*. Subsequently, the LAF measured at position *i* in subclone *j* is computed for every element in *Q* (formula in S1 Text).

Under the assumption that subclone *j* is derived from its parent in the current estimate of the tree $\hat{T}$, not all alleles are equally likely to occur. For example, in case a subclone with 4 copies (AABB) is transformed into a subclone with 3 copies, it is more likely to result in ABB, which only requires a loss of one A allele, than BBB, which would require a loss of two A alleles and a gain of one B allele. To quantify this, we assume that the probability of observing *A*_*i*,*j*_ depends on *A*_*i*,*p*(*j*)_, where *p(j)* denotes the parent of subclone *j*, which is provided in $\hat{T}$, as follows:

$$\begin{array}{c} P\left(A_{i,j}|A_{i,p(j)}\right)=\frac{\frac{1}{ED(A_{i,j},A_{i,p(j)})+1}}{\sum_{q}^{Q}\frac{1}{ED(q,A_{i,p(j)})+1}} \end{array}$$ (4)

Here, the event distance (ED) is computed as the total number of alleles that are different between the parent and the subclone at position *i*. A distance of one is counted for every loss or gain of an allele. The total event distance is computed as the sum of the event distance at every position. $P(A_{i,j}|A_{i,p(j)})$ is normalized based on the event distance to all other alleles in the set *Q*. A pseudocount of one is added to avoid divisions by zero. In conclusion, the event distance allows us to distinguish between for example AB or AABB, which both result in the same LAF measurement.

Following a previously published model, we assume that sequencing noise follows a Gaussian distribution [35]. This assumption requires that the sequencing depth is larger than 1000x. We model the overall probability distribution $P(LAF_{i,j}|C_{i,j},\mu ,\hat{T})$ as a Gaussian mixture model (detailed in S1 Text, see Fig 1D), where the means are equal to the LAFs resulting from each allele combination in *Q*, and the noise component is estimated from the LAF measurements in the normal samples of our real TGCC dataset. The interval of the distribution is limited between 0 and 0.5 to adequately model LAF measurements.

So far, we have only considered a single position *i* and ignored the fact that a horizontal dependency exists between adjacent measurement positions. To incorporate this dependency, we calculate $P(\vec{LAF_{c}}|\vec{C_{c}},\mu ,\hat{T})$. $\vec{C_{c}}$ is a submatrix of $\vec{C}$, containing $\vec{C_{i,j}}$, $\vec{C_{i+1,j}}$, $\vec{C_{i,p(j)}}$ and $\vec{C_{i+1,p(j)}}$ (see Fig 1F and S3 Fig for a detailed example). $\vec{LAF_{c}}$ is a submatrix of $\vec{LAF}$, containing the LAF measurements corresponding to the positions in $\vec{C_{c}}$. $P(LAF_{i,j}|C_{i,j},\mu ,\hat{T})$ is first computed for each copy number in $\vec{C_{c}}$ individually, which are then multiplied to compute $P(\vec{LAF_{c}}|\vec{C_{c}},\mu ,\hat{T})$. Starting from the first two LAF measurement positions, $\vec{C_{c}}$ is iteratively shifted across $\vec{C}$ one position at a time. $P(\vec{LAF}|\vec{C},\mu ,\hat{T})$ is calculated by taking the product of all $P(\vec{LAF_{c}}|\vec{C_{c}},\mu ,\hat{T})$.

#### Computing $P(\vec{C}|\;\hat{T})$

Next, we aim to assign a probability to observing a sequence of copy numbers $\vec{C_{j}}$ in a tumor subclone *j* given $\hat{T}$. We note that the alleles are more informative for evolutionary distance than the copy numbers (see S7 Fig and S1 Text). For instance, if the copy number is 2 in two subclones, we may conclude that the subclones are the same at this position. However, the underlying alleles could be AB and BB, in which case the evolutionary distance is nonzero.

To incorporate the allelic evolutionary distance in the calculation of $P(\vec{C}|\hat{T})$, we can sum the probability of all alleles that can be generated for a $\vec{C_{i,j}}$ as:

$$\begin{array}{c} P\left(C_{i,j}|\hat{T}\right)=\sum_{q\subset Q}P\left(q|\hat{T}\right) \end{array}$$ (5)

However, from computing $P(\vec{LAF}|\vec{C},\mu ,\hat{T})$ we already know that one element in *Q* is much more likely than others given our LAF measurements. Thus, we reason that it is possible to approximate $P(C_{i,j}|\hat{T})$ with the probability of the most likely alleles.

To compute $P(\vec{C}|\;\hat{T})$, we first obtain the most likely alleles corresponding to $\vec{C_{c}}$ (described in Section “Deriving the most likely $\vec{A}$ from a combination of $\vec{C}$ and *μ*”). For these alleles, the Finite State Transducer (FST) shown in Fig 1E is used to compute the event distance that incorporates the horizontal dependency. The FST is used in the MEDICC algorithm for a similar purpose [13]. In the FST, a distance of one is counted for every loss or gain of an allele. In addition, no penalty is given when alleles at adjacent AF measurement positions are affected by the same event. $P(\vec{C}|\;\hat{T})$ is calculated as the product of the event distance computed for each $\vec{C_{c}}$ using the FST. Since $P(\vec{LAF}|\vec{C},\mu ,\hat{T})$ and the event distance are inversely proportional, $P(\vec{C}|\;\hat{T})$ is computed as the reciprocal of the total event distance for $\vec{C}$. Examples of this step are illustrated in Fig 1G and S3 Fig.

#### Maximizing $P(\vec{C},\mu |\vec{LAF},\hat{T})$

Finally, the C-step is completed by inferring a combination of $\vec{C}$ and *μ* for which $P(\vec{C},\mu |\vec{LAF},\hat{T})$ is maximized. To achieve this, we exhaustively evaluate the values of *μ* between 0 and 1 in steps of 0.01. For every *μ*, we vary each copy number in $\vec{C_{c}}$ from a predefined kmin to kmax and select the copy numbers that maximize $P\left(\vec{LAF_{c}}|\vec{C_{c}},\mu ,\hat{T}\right)P\left(\vec{C_{c}}|\;\hat{T}\right)$. $P(\vec{C},\mu |\vec{LAF},\hat{T})$ is computed by taking the product of every $P\left(\vec{LAF_{c}}|\vec{C_{c}},\mu ,\hat{T}\right)P\left(\vec{C_{c}}|\;\hat{T}\right)$. The $\vec{C}$ and *μ* that overall maximize $P(\vec{C},\mu |\vec{LAF},\hat{T})$ are selected as the optimal solution. A more detailed example of how $P(\vec{C},\mu |\vec{LAF},\hat{T})$ is computed for one $\hat{C_{c}}$ is provided in S3 Fig.

#### Deriving the most likely $\vec{A}$ from a combination of $\vec{C}$ and *μ*

To derive the alleles most likely corresponding to a LAF measurement, we define a threshold at the average value between each adjacent LAF measurement in $P(\vec{LAF}|\vec{C},\mu ,\hat{T})$ (Fig 1D). We note that our model is unable to differentiate between the alleles AA and BB. As a result of the low abundance of proximate measurements generated with targeted sequencing, it is not possible to accurately phase alleles. Thus, when computing the horizontal dependency, there is no guarantee that allele A at position *i* is on the same haplotype as allele A at position *i+1*. Therefore, the method will always select the combination with the highest number of B alleles in such ambiguous scenarios.

### T-step

#### Reconstructing *T*

**Fig 2:**
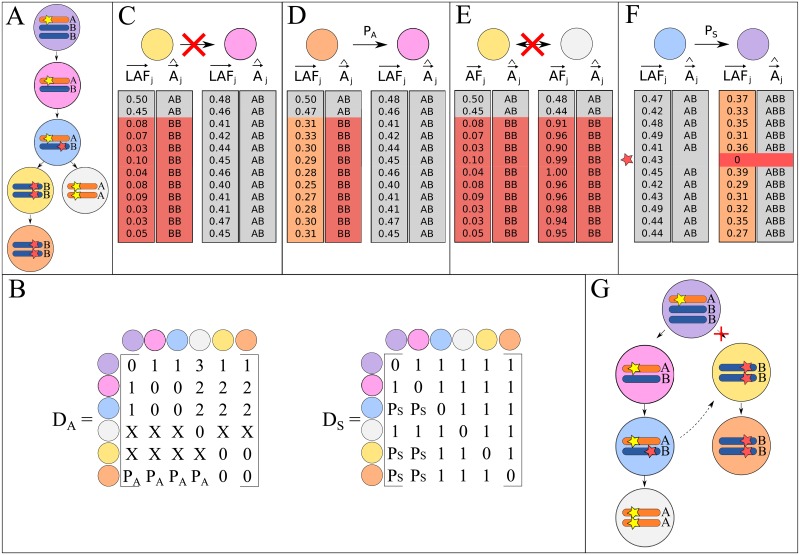
(A) Example of the true *T* for 6 hypothetical subclones. (B) Distance matrices reconstructed from the event distance based on alleles (*D*_*A*_) and somatic SNVs (*D*_*S*_). ‘X’ indicates that a subclone cannot be the parent of another subclone. (C) and (D) Edges can be restricted or penalized based on LOH. Each row in the matrix represents a measurement position on the genome. The measured LAF and the $\hat{\vec{A}}$ inferred by TargetClone are shown in separate columns. A grey color represents a balanced situation, orange allelic imbalance, and red LOH. The first two measurements are not shown in the tree in panel (A). (E) If different parental alleles are lost, edges can be restricted. The ground truth alleles are AA in the grey subclone, but TargetClone will report the alleles as BB. (F) Edges can be penalized if the loss of somatic SNVs is unlikely. (G) Example of the MSA for the subclones shown in (A). The red cross indicates that an edge in the MSA is removed when resolving the ISA. The dashed line indicates a newly added edge after resolving the ISA.

To reconstruct the evolutionary tree *T* of sampled subclones using the inferred alleles (see Fig 2A for an example of *T*), we assume that the optimal tree has a minimum event distance between all subclones in the tumor, and thus corresponds to the minimum spanning arborescence (MSA) [13]. Sample by sample distance matrices are generated to describe to relationship between each pair of subclones. The distance matrix *D*_*A*_ (Fig 2B) is constructed by calculating the allelic event distance between all combinations of subclones using the FST (Fig 1E). Distance matrix *D*_*S*_ describes the distances based on somatic SNVs, and initially only contains a value of 1 to indicate that a parental relationship is possible. The values in both matrices may be penalized as discussed below. As the distances based on alleles and somatic SNVs must both agree on a relation between subclones, matrices *D*_*A*_ and *D*_*S*_ are multiplied to generate the final distance matrix *D*_*F*_. This final distance matrix is used as input to Edmonds’ algorithm, which infers an MSA [36].

The inferred alleles and the measured somatic SNVs provide additional information that we can use to restrict or resolve the relations between subclones in the tree.

**Restricting and penalizing $\hat{T}$ based on LOH**—Edges in $\hat{T}$ can be restricted based on regions with loss of heterozygosity (LOH), as re-gaining lost alleles is highly unlikely (Fig 2C). By default, we consider LOH to be present in a subclone when at least 10 consecutive LAF measurements are smaller than 0.3, and either of the parental alleles has been estimated as lost in $\hat{\vec{A}}$. Both settings can be changed by the user if necessary. In Fig 2D, an example is shown where the LAF measurements are not smaller than 0.3. In this scenario, we cannot confidently decide whether the region shows LOH and that the percentage of normal admixture is high, or if $\hat{\vec{A}}$ is incorrect. Thus, rather than restricting an edge between the subclones, we add a penalty *P*_*A*_ to the current value in *D*_*A*_.

**Restricting $\hat{T}$ based on the loss of different parental alleles**—Relations between subclones can also be restricted based on AF measurements. If two subclones contain LOH and have lost a different parental allele, the first subclone cannot be the parent of the second subclone and vice versa (Fig 2E). Although TargetClone cannot distinguish between the parental alleles, we consider a different parental allele to be lost when the AF is lower or higher than 0.1 and 0.9, respectively. These default values may be changed by the user.

**Restricting $\hat{T}$ based on somatic SNVs**—The edges between subclones can also be restricted based on the measured somatic SNVs. One assumption is that somatic SNVs are typically not lost, unless the allele that these are present on is also lost. If no evidence is present of a lost allele (Fig 2F), we assign a penalty *P*_*S*_ to these types of relations.

**Resolving the ISA by editing the MSA**—It often occurs that a MSA is obtained that violates the ISA (Fig 2G). Based on the minimum distance assumption, we reason that it is possible to use the MSA as a starting point, and perform edit operations until the ISA is no longer violated. To this end, under the assumption that subclones should differ minimally from their parents, we expect that the edge in which the most somatic SNVs are introduced is most likely spurious. In case of a tie, a random edge is selected from the spurious edges. Our method iteratively removes the selected edge from the tree and re-runs Edmonds’ algorithm on all remaining possible edges between all subclones to infer a new tree until the ISA is resolved. By default, 50 updated trees are generated from the starting MSA, from which the tree with the lowest allelic distance between all subclones is selected as the final solution. 50 trees are explored to prevent the method from getting stuck in a local maximum and thus increases the likelihood that the method generates the same tree for each run.

There are situations in which the ISA may not hold, for example in scenarios where somatic SNVs are drivers of tumor evolution [37], and are therefore expected to independently recur in independent subclones. For this reason, if the ISA cannot be resolved, the edited tree with the fewest violations of the ISA and lowest total distance will be reported. The total distance is computed by taking the sum of all edge weights in the tree, which are obtained from the final distance matrix *D*_*F*_. Furthermore, we allow the user to select somatic SNVs to be excluded from analysis with TargetClone. Furthermore, The final top 10 trees are visualized using the Bokeh plotting library [38], as described in S1 Text.

### Simulation data

#### Generation of simulation data

Starting from a healthy, diploid cell, we formed subclones with new somatic SNVs and CNVs for 4 rounds (see S1 Text and S4 Fig A for details on how the simulated data is created). On average, 5 samples are generated, including the healthy cell. The relations between the subclones and precursors decide the ground truth *T*. All generated subclones and precursors were sampled, which were assigned the same tumor fraction. Selecting the same tumor fraction allows us to additionally test what the effect is of each tumor fraction individually on the performance. In total, per sample, 500 AF/LAF and 50 somatic SNV measurements were generated based on the simulated somatic SNV and CNV profiles to model targeted sequencing data. These measurements were assigned randomly to each chromosome arm, but each chromosome arm on average has an equal number of SNPs.

In our TGCC dataset, we assumed that our sequencing noise is Gaussian distributed, and estimated the standard deviation to be 0.02 in our reference samples. Thus, we selected noise levels of 0.005, 0.01, 0.015, 0.02, 0.025 and 0.03 to represent realistic levels of noise, and 0, 0.04, 0.06, 0.08 and 0.1 representing more extreme sequencing noise levels to test the limits of the method. By default, TargetClone uses a diploid precursor in the initial tree $\hat{T1}$. In Section “TargetClone yields high-quality trees”, we also explore the effect on the results if a random precursor ploidy is used.

All results on simulated data discussed in the main text refers to the data generated as described in this section. In addition, we also generated a more realistic simulation dataset closely modelling TGCC data. The generation of this data and related results are discussed in S1 Text.

#### Computing the error on the simulation data

*E*_*C*_ is the error of $\hat{\vec{C}}$, which is computed as the absolute distance with respect to the true $\vec{C}$, which is normalized for the size of $\vec{C}$. The error in $\hat{\vec{A}}$, *E*_*A*_, is defined as the average event distance between $\vec{A}$ and $\hat{\vec{A}}$ across all positions. The horizontal dependency is not taken into account in the calculation of the error, as we wish to score the error at each position in $\hat{\vec{A}}$ individually. *E*_*μ*_, which is the error of $\hat{\mu }$, is computed as the mean absolute error with respect to *μ*. To test how well ancestry relationships are reconstructed in our trees, we investigated how often parent-child relations were inferred incorrectly. For each pair of samples, we computed how often a parent-child relationship was absent in the inferred tree (false negative) and we also computed how often parent-child relationships were present in the inferred tree, but not in the ground truth tree (false positive). The total tree error, *E*_*T*_, is calculated as the sum of the number of false positives and false negatives, which is normalized by the total number of sample pairs. The error calculation formulas are provided in S1 Text.

## Results

### Simulation data results

**Fig 3:**
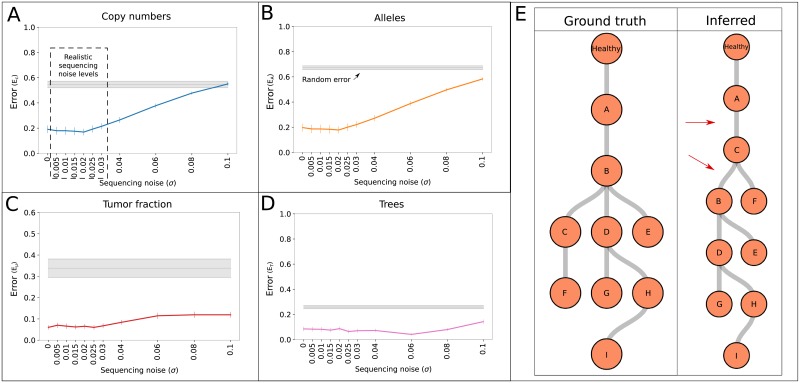
(A-D) The error of inferring $\vec{C}$, $\vec{A}$, *μ* and *T* as a function of sequencing noise. For every noise level, the mean of the error and 95% confidence interval are reported across 101 simulated datasets, each with a unique *μ* between 0 and 1. The grey shaded areas represent the mean of the error and 95% confidence intervals in 101 simulated datasets where random AF and SNV measurements were selected. (E) Example of a simulated tree (ground truth) compared to the tree inferred by TargetClone. The red arrows indicate incorrectly placed edges.

To test TargetClone on realistic data for which the ground truth is known, we generated 101 simulation datasets as described in the methods section. Fig 3A–3D shows the error of inferring $\vec{C}$, $\vec{A}$, *μ* and *T* across the simulations as a function of sequencing noise. The grey shaded areas indicate the mean of the error and 95% confidence interval obtained by running TargetClone on 101 simulation datasets with random data. In each random dataset, a different *μ* between 0 and 1 was selected. The same AF and somatic SNV measurement positions as in the non-random simulation datasets were selected. At each AF and somatic SNV measurement position, a random AF and somatic SNV measurement between 0 and 1 was selected. As a result, they provide a reference error rate based on the performance of the method by random chance.

#### TargetClone yields high-quality trees

The error profile of $\hat{\vec{C}}$ and $\hat{\vec{A}}$ reveals that the inference of copy numbers and alleles is highly accurate, in particular in the range of realistic sequencing noise levels. The error rate increases as sequencing noise increases, ultimately reaching the error rate expected by random chance for very high noise levels. The inference of *μ* is more robust to sequencing noise, indicating that the LAF measurements are still sufficiently informative to estimate *μ* correctly despite the increase in noise level. Noteably, the error rate of predicting *μ* correctly by random chance has larger confidence intervals, which results from *μ* estimates always being in the range of 0.7–0.91 in each simulated dataset. Thus, since all *μ* between 0 and 1 are tested, the error decreases as the true *μ* of the dataset increases, particularly showing low error rates when the true *μ* lies within this range of estimated *μ*.

In S5 Fig we show that re-running TargetClone yields approximately the same results.

To assess the quality of the solution for different initializations, we repeated the optimization for random starting trees ($\hat{T1}$). In these random trees, the relationships between all subclones were selected randomly. For each subclone that was selected as a parent in the random tree, the ploidy of the alleles were selected randomly, which are normally diploid. S6 Fig shows that very similar results are obtained, demonstrating robustness for the initialization of the optimization.

In S7 Fig and S1 Text, we show that combining alleles and somatic SNVs, together with resolving the ISA, yields the largest benefit in reconstructing the trees as compared to when the trees are reconstructed with alleles, copy numbers or somatic SNVs individually. We additionally show that the number and distribution of SNP measurements and the number of measured SNVs does not significantly affect the quality of the inferred copy numbers, alleles and tumor fraction (S1 Text, S8 and S9 Figs).

Fig 3E shows an inferred tree with two differences with respect to the ground truth tree. Relations B-C and B-F are missed in the inferred tree (false negatives), and relations C-B, C-E, C-D, C-G, C-H and C-I are introduced (false positives). The total number of sample pairs in this tree is 45, and thus the error rate of this tree would be 8/45 = 0.18. For realistic noise levels, the mean tree error obtained by TargetClone is approximately 0.1. (Fig 3D, see S10 Fig for a figure showing the false positive and false negative rates independently). Clearly, trees with so few errors are useful to investigate subclonal development and yield similar conclusions, despite the few differences with respect to the ground truth.

#### Tumor fraction is a determinant of error rate

**Fig 4:**
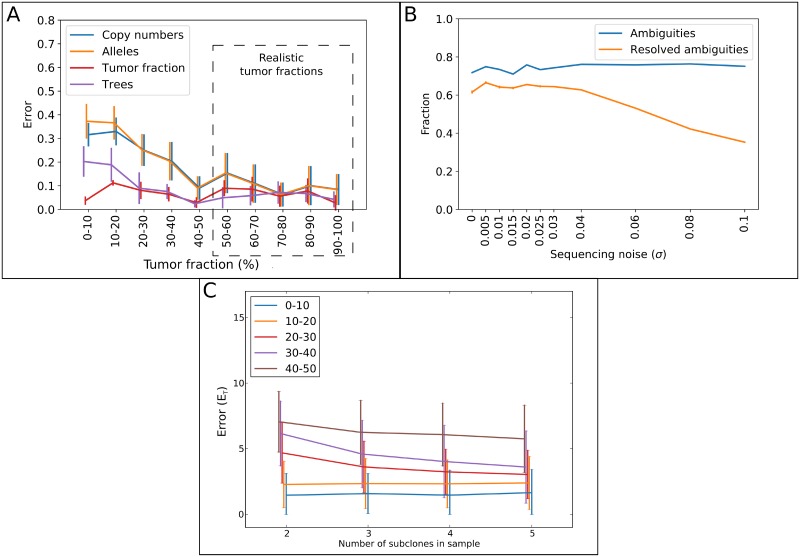
(A) Mean of the error rates and 95% confidence intervals as a function of different tumor fractions at a sequencing noise level of 0.02. Every *μ* was tested once. (B) The blue line shows the average fraction of ambiguous alleles that are present in 101 simulated datasets, each with a different tumor fraction between 0 and 1. The orange line indicates the mean and 95% confidence intervals of resolved ambiguities, normalized by the size of $\hat{\vec{A}}$. (C) Mean of the tree reconstruction error rates and 95% confidence intervals as a function of the number of subclones in the sample. A total of 100 simulations were performed for each number of subclones, for each of which a noise level of 0.02 and *μ* of 0.9 was selected. Each line shows the total percentage of the contaminating minor subclones in each sample. Every contamination percentage within the shown range was tested once.

Fig 4A and S11 Fig show that robust performance is measured at realistic and common tumor fractions in microdissected samples [39–41]. For lower tumor fractions, a higher error rate for $\hat{\vec{C}}$ and $\hat{\vec{A}}$ is obtained than for high tumor fractions. Thus, a high amount of healthy cell contamination, which pushes the LAF measurements towards 0.5, obfuscates information about the tumor subclone. Furthermore, the estimation of *T* is more accurate at realistic tumor fractions. In short, obtaining high sample tumor fractions benefits subclonal reconstruction accuracy, further justifying the advantage of microdissections.

#### Ambiguous alleles can be correctly resolved

Many combinations of alleles and tumor fraction give rise to the same LAF. For example, both allele combinations AABB and AB for a *μ* of 0.5 give rise to a LAF measurement of 0.5. Thus, the exact allele at such a position is impossible to derive based on the LAF measurement of that position alone, and hence is considered ambiguous. In our simulation data, for which the ground truth alleles are known, on average 75% of simulated alleles are ambiguous (Fig 4B).

To investigate the effect of these ambiguities, we aimed to demonstrate how well our method is able to resolve the correct allele. Interestingly, TargetClone is able to infer the correct alleles for around 80% of these ambiguous positions. In part this is due to the assumption of vertical dependency, which ensures alleles in $\vec{A}$ are chosen that minimize the event distance to its parental subclone. To investigate the importance of the presence of the vertical dependency in a dataset for resolving ambiguities, we computed how often the allelic event distance between a subclone and its parent is larger than the distance to any other subclone in a tree. We correlated these values with the number of unresolved ambiguities in the same subclones, and found a Pearson correlation coefficient of 0.23. Thus, we conclude that the ability of TargetClone to resolve ambiguities is not significantly affected by cases where the vertical dependency between the subclones is not as strong.

Second, LOH regions are informative of *μ*, and as a result greatly restrict the number of possible alleles. For example, a LAF of approximately 0.33 can be measured in a sample with a tumor subclone with alleles ABB or ABBB at one position with tumor fractions of 0.9 and 0.5, respectively. However, if LOH is present at another position, where a LAF of for example 0.09 is measured, the ambiguity is resolved, as this LAF measurement cannot be obtained with a tumor fraction of 0.5 at realistic sequencing noise levels.

It is also important to note that errors in $\hat{\vec{A}}$ resulting from measurement ambiguities may not necessarily negatively affect $\hat{T}$. For example, if the measured LAF is 0.5, it may be explained by multiple combinations of $\vec{A}$ and *μ*, such as AABB or AB with a *μ* of 0.5. However, the event distance between two subclones does not change if the alleles are inferred to be AB in both subclones instead of AABB, and thus, no effect is observed on $\hat{T}$ even though an error is made in $\hat{\vec{A}}$. In conclusion, we showed that the assumptions made in our model are sufficient to resolve measurement ambiguities.

#### TargetClone can reconstruct trees for polyclonal samples

To investigate the effect of multiple co-existing subclones in a sample on the performance of TargetClone, we generated additional simulation datasets with a sequencing noise level of 0.02. The *μ* of these datasets was fixed at 0.9. As is shown in Fig 4A, the error rates of the method are low with relatively small confidence intervals at this *μ*, thus allowing us to test the influence of polyclonality at a realistic *μ* that itself does not largely influence the results. Each simulated sample consists of one major subclone (at least 50% of the total tumor content), and increasing levels of contamination from random other subclones from the same tumor. We observe that the inference of $\vec{C}$, $\vec{A}$, *μ* and *T* is robust to increasing number of subclones (Fig 4C and S12 Fig). For *T*, the error rate at a contamination level between 40 and 50% is as low in samples containing 5 subclones (4 minor subclones contaminating around 10%) as in samples containing 2 subclones (major and minor subclone both present in around 50%). Thus, reducing the total level of contaminating minor subclones yields higher performance improvement than reducing the number of contaminating subclones, which is consistent with our assumption that samples require one major tumor subclone. It has been shown that in practice, microdissected samples can most often indeed contain one major subclone, with relatively small contamination of minor subclones [23].

### Real data results

We applied TargetClone to samples from 4 patients with TGCC (NS) with intrinsic resistance to chemotherapy. Multiple histological components were microdissected from each tumor (S2 Fig), which were subjected to targeted sequencing [23]. In total, each patient has 9, 6, 18 and 10 samples, with 15, 43, 32 and 31 measured somatic SNVs, and 427, 420, 435 and 407 AF measurements (in patient T6107, T6108, T3209 and T1382, respectively).

**Fig 5:**
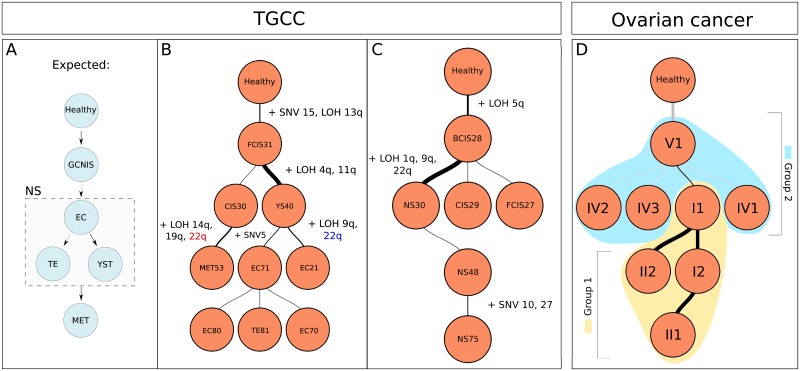
(A) Expected development of TGCC based on knowledge described in literature. (B) Tree reconstructed by TargetClone for T6107. (C) Tree reconstructed by TargetClone for T618. A few events have been annotated to show the relations between samples. In **(B)**, LOH at chromosome 22q is colored in blue and red to indicate that a different parental allele has been lost. A thicker line indicates that a larger number of events is introduced in the subclone. (D) Tree reconstructed by TargetClone for P1 of the ovarian cancer dataset. The two sample groups are placed in two clusters, as highlighted in yellow and blue. A description of how the trees are visualized can be found in S1 Text.

The sequencing depth is 1000x on average. Since no ground truth is known for the development of these specific tumors, the results are compared to knowledge previously described in literature (Fig 5A). In summary, TGCC are expected to start development from a tetraploid precursor GCNIS (referred to as CIS in sample names). GCNIS can further develop into NS, which may consist of multiple histological components, including Embryonal Carcinoma (EC), Yolk Sac Tumor (YST), Teratoma (TE) and Embryonal Bodies (EB) [33, 42, 43]. It has been shown that TE and YST can only develop from EC [33, 44, 45].

Based on this knowledge, we defined that in the initial tree $\hat{T1}$, the parent of every subclone is a tetraploid cell, rather than a healthy, diploid cell. Fig 5 shows the inferred subclonal evolution tree for 2 patients, T6107 (Fig 5B) and T618 (Fig 5C). The trees reconstructed for the other 2 patients are shown in S13A Fig (T3209) and S13B Fig (T1382).

On average, the trees for the real data were reconstructed in 30 minutes on 1 CPU core with 12GB of memory.

#### Case 1: T6107

Fig 5B shows that the predicted evolution tree of T6107 closely resembles the predefined expectations in Fig 5A. Interestingly, samples MET53 and EC21 are correctly placed in different branches. Both samples contain LOH at chromosome 22q, but from the AF it becomes clear that a different parental allele has been lost and thus there exists no direct relation between these samples. Sample MET53 is predicted to have formed from the early precursor CIS30. Sample MET53 lacks all somatic SNVs that have been measured in samples other than CIS30 and FCIS31, and contains a unique pattern of LOH.

The placement of sample YS40 does not correspond to the expectations, as YST can only originate from EC. Nevertheless, YS40 lacks one somatic SNV compared to the EC and TE samples, and thus the ISA cannot be resolved if YS40 is placed elsewhere. As an explanation, it is likely that an unsampled EC subclone existed after FCIS31, which gave rise to YS40, EC21 and the other EC and TE samples.

#### Case 2: T618

Fig 5C shows the inferred tree for patient T618. CIS is expected to develop into BCIS, which in turn develops into FCIS. FCIS can then develop further into the histological components of NS. From the data, we note an indication that a different parental allele may have been lost at chromosomes 11 and 22 in BCIS28 and FCIS27 and the primary tumor (NS). Thus, it is likely that an unsampled precursor exists that branched into CIS29, FCIS27 and into BCIS28, which then further developed into NS. In our result, sample CIS29 is instead predicted to develop from BCIS28 for two reasons. First of all, LOH is not detected by the model on chromosomes 11 and 22 in BCIS28 and FCIS27 as no 10 consecutive measurements support that LOH. Finally, CIS29 contains additional somatic SNVs that have not been measured in FCIS27 and BCIS28. The primary tumor (NS) has acquired additional mutations, and independent runs of the primary tumor sample (NS48, NS30, NS75) are placed at the bottom of the tree as expected.

#### The choice of precursor ploidy influences the quality of $\hat{T}$

No proof yet exists for the assumption that TGCC are initiated by genome duplication. To further investigate this question, we also reconstructed evolutionary trees for our TGCC cases with an assumed diploid precursor (S14 Fig). The reconstructed tree for T3209 does not follow the biological expectations very well, as sample TE86 cannot be the precursor of EC samples. The total distance between all subclones is higher in the trees generated with a diploid precursor (294, 1054, 3473, 1213 with a diploid precursor and 227, 657, 578, 943 with tetraploid precursor in T618, T6107, T1382 and T3209, respectively). Although the tree for T1382 could not be reliably reconstructed due to high numbers of unsampled subclones and high levels of sequencing noise, and for T618 only a limited number of samples was sequenced, more support is obtained for the assumption that TGCC develop after a duplication of the diploid genome. Although no hard conclusions about precursor ploidy can be drawn from this limited set of samples, the observation that higher distances are obtained and that biological assumptions can be violated when a different initial ploidy is selected, highlights the importance of choosing the correct precursor ploidy. If the ploidy of the precursor is not known, we recommend selecting the ploidy for which the minimum total distance between all subclones in the final tree is reported.

#### A comparison of TargetClone to existing methods on targeted sequencing data

Finally, we aimed to determine how TargetClone compares to existing tools to reconstruct subclonal evolution trees on targeted sequencing data with microdissected samples. This comparison is challenging, as no method exists that is specifically designed to work with targeted sequencing data from microdissected samples. For this reason, we performed the comparisons under the assumption that one tumor subclone is present per sample. In our comparison we included PhyloWGS, which is currently the only method that combines SNVs and CNVs to infer evolutionary trees (see S1 Fig), thus making it the most suitable method to compare with TargetClone. Second, we selected the SNV-only method LICHeE, which infers trees from cellular prevalences estimated with PyClone [46]. Third, we ran LICHeE directly on VAFs to demonstrate the effect of including cellular prevalences. Details on the settings of these methods are described in S1 Text.

The trees inferred by PhyloWGS, PyClone + LICHeE and LICHeE are provided in S19–S21 Figs. Inspection of these trees (described in detail in S1 Text) reveals that none of these trees match with the established knowledge on TGCC development. PhyloWGS appears to miss many subclones and LICHeE fails to detect important relations between subclones that are apparent from LOH patterns. Notably, all of the relations missed by PhyloWGS, PyClone and LICHeE were captured by TargetClone, with the exception of T1382, for which we cannot make a clear statement about the quality of the inferred tree due to the large number of unsampled subclones. Thus, we conclude that the analysis of targeted sequencing data is a difficult task that is not well dealt with by existing methodology. TargetClone, which is tailored to deal with targeted sequencing data, does provide insightful trees containing evolutionary relations that are missed by the currently available tools. These findings are supported by our comparison of TargetClone with existing methods on simulated targeted sequencing data, which is discussed in S1 Text.

#### TargetClone applied to an ovarian cancer dataset

To determine how well TargetClone performs on another tumor type, we applied it to 8 samples taken from physically separated tumor sites in the abdomen of an ovarian cancer patient [34]. Although these samples were not microdissected, it is shown in the original paper that there exist two sample groups with independent clusters of mutations, and a number of samples contain private mutations with VAF > 0.1. Based on these observations, we expect that the topographic sampling sufficiently reduces heterogeneity to major clones, thus providing an additional test case for TargetClone.

In total, 58 somatic SNVs were measured with targeted sequencing and the AF was measured at approximately 300000 SNP positions using a SNP array. It was previously observed that sample group 1 (I1, I2, II1, II2) and 2 (IV1, IV2, IV3 and V1) contain two clusters of mutations that are mutually exclusive, and we thus expect TargetClone to identify that these groups to have independent origins. Noteably, sample group 2 shares a number of mutations with group 1. However, the low allele frequencies of these mutations point to likely contamination with other subclones.

TargetClone reconstructs a tree in which both groups are clustered together, matching our expectations (Fig 5C). In conclusion, TargetClone provides useful insight into the development of this tumor, even though the data consists of non-microdissected heterogeneous samples.

#### Comparing TargetClone with existing whole genome sequencing-based methods

Finally, we aimed to determine the benefits of running TargetClone on targeted sequencing data instead of using existing tools applied to WGS data. To do so, we compared the results of TargetClone on SNP array and targeted sequencing data (Fig 5C) with the result obtained by PhyloWGS, PyClone coupled with LICHeE (S25 Fig), and LICHeE with VAFs (S26 Fig) on WGS data of our ovarian cancer dataset.

PhyloWGS could not infer a tree. The trees reported by PyClone coupled with LICHeE and LICHeE alone do not capture the relationships between the two sample groups with mutual exclusive mutations (details in S1 Text). These poor results are most likely explained by the low read depth (3X on average) of our WGS dataset. Taken together, we have shown that running TargetClone on targeted sequencing data does not miss information that is captured by applying existing methods on WGS data.

## Discussion

In this article, we described TargetClone, a novel method to infer copy numbers, alleles, the fraction and subclonal evolution trees of tumors from SNP AF and somatic SNVs measured in microdissected samples. We demonstrated on simulation data that our method obtains low error rates for inferring $\vec{C}$, $\vec{A}$, *μ* and *T* at realistic levels of sequencing noise and realistic sample tumor fractions. Furthermore, we show that at approximately 80% of ambiguous LAF measurements the correct alleles are estimated. Existing algorithms always rely on read depth information, either by requiring that somatic SNVs are located in copy number-neutral regions, or by directly using CNVs. We have now demonstrated that in samples that contain at least one major subclone, a combination of somatic SNVs and AFs can be sufficient to accurately reconstruct copy numbers, alleles, fractions and evolutionary trees of tumors. These findings suggest that it is possible to obtain a good insight into subclonal tumor evolution even if read depth information is noisy and biased.

A current limitation of our approach is the assumption that purified samples contain only one tumor subclone. We showed that, in practice, TargetClone is not markedly affected by samples containing more than one subclone, as it still produces trees with few errors up until on average 20% of contamination with minor subclones. Although it has been shown that it is possible to obtain samples with at least one major subclone and limited minor contamination [23], it may not always be known beforehand what the total percentage of contamination in a sample is. In the future, single-cell sequencing may mitigate this limitation.

We also note that there are some limitations to the use of the FST. In short, the FST does not model biological constraints, allowing for example the re-gain of alleles when inferring the most likely alleles in a subclone. To overcome this, our model limits relations between subclones when inferring *T* if there is evidence in $\hat{\vec{A}}$ that alleles would require to be re-gained. A potential alternative would be to adapt the FST to include restrictions based on biological constraints, removing the need for ad-hoc corrections. However, we argue that enforcing such restrictions at an early stage in the model would reduce the potential to estimate $\vec{A}$ correctly if many subclones were unsampled. Since the model infers alleles that minimizes the event distance, in such scenarios the inferred alleles will be more similar between subclones, misrepresenting the actual underlying allelic composition.

TargetClone currently does not scale to whole exome sequencing data, as our method infers $\vec{C}$ and $\vec{A}$ for every SNP individually. Runtimes can be reduced by a pre-segmentation of SNPs into regions with equal AF. Furthermore, resolving the ISA will become more difficult when a higher number of, potentially noisy, somatic SNVs are measured. We therefore recommend to either exclude somatic SNVs with low confidence and quality from reconstructing the ISA, which is provided as an option in TargetClone, or cluster the somatic SNVs into groups of somatic SNVs that are shared or absent across samples to reduce the influence of noise.

We employed TargetClone on four TGCC cases and one ovarian cancer case to study their subclonal evolution. We found that the inferred trees are mostly consistent with our expectations of the development of these tumors. Thus, the reconstructed trees are helpful to study relations between tumor subclones, which can assist in gaining insight into development and progression of the tumor.

## Supporting information

S1 Fig Existing methods that can decompose subclones from mixed samples and/or reconstruct subclonal evolution trees. For each method, it is listed which data types are used and if trees are reconstructed or not. As this paper focuses on mixed samples, single-cell-based methods have been omitted from this overview.
(EPS)

S2 Fig Example of microdissections applied to our real data case of testicular germ cell cancer (nonseminoma) [23]. (A) H&E staining (original magnification x 2) of a section from T3209 showing the complexity of this primary testicular mixed germ cell tumor. The major tumor component in this section is solid and glandular embryonal carcinoma (EC), with in between highly vascular mesenchymal teratomatous tissue with scattered epithelial structures (T), small areas of yolk sac tumor (YST) and trophoblastic giant cells (TGC). Larger areas of teratoma and yolk sac tumor are present in adjacent sections of this case. A so-called embryoid body (EB), comparable to a day 10-human embryo, derived from a single embryonal carcinoma cell, is present in the encircled area, and shown at higher magnification in panel (B). Pictures taken from PALM-assisted purification of tumor cells from frozen tissue sections, visualized by direct alkaline phosphatase reactivity, are shown in panels (C) and (D) (before purification), and (E) (during purification).
(EPS)

S3 FigToy example calculation of $P(\vec{C},\mu |\vec{LAF},\hat{T})$ for one $\vec{C_{c}}$, thus with two samples and two measurements.(A) We start with estimates of $\vec{C_{c}}$ and *μ* given the LAF measurements and an initial tree where the parent of each sample is diploid. (B) Computation of $P(\vec{LAF}|\vec{C},\mu ,\hat{T})$ for one $\vec{C_{c}}$. In step 1, we compute the probability distribution for the current *μ* estimates, which are 1 and 0.5, and each copy number in $\vec{C_{c}}$, which are 2 and 1, respectively. An example of how the probabilities are computed is detailed in Fig 1. In step 2, we obtain the actual probabilities that would be assigned to the measured LAF for these *C* in $\vec{C_{c}}$ and *μ*. All four values in $\vec{C_{c}}$ are multiplied to obtain the final probabilities. (C) Computation of $P(\vec{C}|\hat{T})$ for one $\vec{C_{c}}$. In step 3, we use the known LAF measurements to derive from the probability distributions of step 1 what the alleles would be. In step 4, we compute the event distance based on the alleles corresponding to $\vec{C_{c}}$ as derived in step 3. Under the horizontal dependency assumption, the FST will compute an event distance of 1. The total probability is computed as 0.5. (D) In step 5, $P(\vec{C_{c}},\mu |\vec{LAF_{c}},\hat{T})$ is computed by multiplying the probabilities obtained at step 2 and step 4.(EPS)

S4 Fig Generation of simulation data for (A) the generic simulations and (B) the TGCC-based simulations. Unviable subclones are not allowed to continue through further cell divisions. The final remaining subclones at cycle 4 are sampled to generate input for TargetClone.
(EPS)

S5 FigRe-running TargetClone 100 times on the same simulated dataset gives approximately the same results.For each simulation re-run, we computed the difference to the error of all other re-runs, of which the average is reported in the figure. The tumor fractions differ more often between re-runs than $\vec{C}$, $\vec{A}$ and *T*, but the low average difference indicates that this happens in a minimum number of re-runs.(EPS)

S6 FigMean of the error and 95% confidence intervals for $\hat{\vec{C}}$, $\hat{\vec{A}}$, $\hat{\mu }$ and $\hat{T}$ in the simulated datasets where a random tree was used as $\hat{T1}$.Only realistic noise levels are shown. At every noise level, 101 simulated datasets were generated, each wth a unique *μ* between 0 and 1.(EPS)

S7 Fig The mean of the tree reconstruction error and 95% confidence intervals when different data types are used to reconstruct the distance matrices in comparison to the error obtained by TargetClone. A total of 101 simulated datasets were tested, each with a different *μ* between 0 and 1.
(EPS)

S8 Fig Increase in the number of SNPs to show the effect of having fewer or more LAF measurements. For each number of SNPs 100 simulated datasets were generated with a noise level of 0.02 and a *μ* of 0.9. Because we measured the error rate with a *μ* of 0.9, *T*_*e*_ is significantly lower than *T*_*e*_ in Fig 3D.
(EPS)

S9 Fig The error rates obtained when the number of somatic SNV measurements are increased. For each number of SNVs, 100 simulated datasets were generated with a noise level of 0.02 and a *μ* of 0.9.
(EPS)

S10 Fig The false positive and false negative rates for the trees inferred in our simulation data. The combined FPR and FNR is shown in Fig 3D.
(EPS)

S11 FigError rates for $\hat{\vec{C}}$, $\hat{\vec{A}}$, $\hat{\mu }$ and $\hat{T}$ as a function of *μ* in the simulated datasets.Every simulated dataset has one unique *μ* between 0 and 1. The mean of the error and 95% confidence intervals are reported in bins of *μ*. The noise levels are shown as separate lines. Not all tested noise levels are shown to improve visualization.(EPS)

S12 FigThe mean error and 95% confidence interval of $\hat{\vec{C}}$, $\hat{\vec{A}}$ and $\hat{\mu }$ as the number of subclones in the sample increases.Each line incidates the total percentage of contamination of the minor subclones in the sample. For each simulated dataset, a *μ* of 0.9 and a noise level of 0.02 was selected.(EPS)

S13 Fig Reconstructed trees for (A) T3209 and (B) T1382 when a tetraploid precursor is used. For T3209, we selected the second best reported tree, as the development of other histological components (other than CIS) from EC75 instead of TE86 matches biological expectation better. For T1382 the ISA could not be resolved and thus the MSA with the fewest ISA violations is reported. All events that are introduced multiple times independently are highlighted in red. A thicker line indicates that a higher number of events is gained in the subclone.
(EPS)

S14 Fig Reconstructed trees for (A) T6107, (B) T618, (C) T3209 and (D) T1382 when a diploid precursor is used. For T1382 the ISA could not be resolved and thus the MSA with the fewest ISA violations is reported. A thicker line indicates that a higher number of events is gained in the subclone.
(EPS)

S15 Fig Segmentation of the corrected read depth sample EC85 of T3209 by CNVKit. (EPS)

S16 Fig Comparison of *μ* estimates of TargetClone to ASCAT and THetA for (A) T3209, (B) T6107, (C) T618 and (D) T1382. (EPS)

S17 FigComparison of $\vec{C}$ estimates of (A) TargetClone to (B) THetA for sample EC70 of T6107. The SNP (AF) and somatic SNV (VAF) measurements of this sample are shown in (C).(EPS)

S18 FigComparison of $\vec{C}$ estimates of (A) TargetClone to (B) THetA for sample TE74 of T3209. The SNP (AF) and somatic SNV (VAF) measurements of this sample are shown in (C). In (B), THetA estimated a copy number of 4301 for chromosome 19, which was left out of this figure.(EPS)

S19 Fig Trees inferred by PhyloWGS for (A) T3209 (B) T618. The most interesting events are annotated in the trees. The order of the somatic SNVs is equal to the order of the somatic SNVs in the original input file and thus corresponds to the events annotated in the trees generated by TargetClone. (A) Samples in subclones: each subclone is present in every sample. (B) Samples in subclones: 1: all samples, 2, 3 and 8: CIS29, FCIS27, NS75. 4, 5 and 6: FCIS27. 7: NS75.
(EPS)

S20 Fig Trees inferred by LICHeE using the cellular prevalences inferred by PyClone for (A) T3209, (B) T6107, (C) T618 and (D) T1382. (EPS)

S21 Fig Trees inferred by LICHeE using the VAF of somatic SNVs for (A) T3209, (B) T6107, (C) T618 and (D) T1382. (EPS)

S22 Fig Mean of the inference error and 95% confidence intervals on the TGCC-based simulations for (A) copy numbers (B) alleles (C) tumor fraction and (D) trees. (EPS)

S23 Fig Schematic representation of trees reconstructed by TargetClone, LICHeE and MEDICC for one simulation dataset. The precursor node indicates the 4N precursor. The numbers on the edges represent the estimated distances between the nodes. The tree reconstructed by LiCHeE correlates negatively with the ground truth as the distances between the precursor and pre-GCNIS nodes are larger than the distances to subclones A, B and C, whereas the ground truth distances are the opposite. A similar pattern is observed for MEDICC.
(EPS)

S24 Fig Correlation of the distance matrices produced by TargetClone, MEDICC and LICHeE with the ranked ground truth distances. (EPS)

S25 Fig Tree inferred by coupling PyClone with LICHeE for our ovarian dataset. (EPS)

S26 Fig Tree inferred by LICHeE for our ovarian dataset. (EPS)

S1 Text Supplemental methods and results. (PDF)
