# Supplementary material for: TargetClone: A multi-sample approach for reconstructing subclonal evolution of tumors
Source: PLoS One. 2018 Nov 29;13(11):e0208002. doi: 10.1371/journal.pone.0208002 (PMC6264523; doi:10.1371/journal.pone.0208002)
Supplement: S1 Text — (PDF) [file pone.0208002.s027.pdf]

TargetClone: a multi-sample approach for reconstructing subclonal evolution of tumors

Supplementary Data

Marleen M. Nieboer[1], Lambert C.J. Dorssers[2], Roy Straver[1], Leendert H.J. Looijenga[2,3], and Jeroen de Ridder[1]

[1]Center for Molecular Medicine, University Medical Center Utrecht, Utrecht, The Netherlands
[2]Department of Pathology, Erasmus MC Cancer Institute, University Medical Center Rotterdam, Rotterdam, The Netherlands
[3]Princess Maxima Center for Pediatric Oncology, Utrecht, The Netherlands

1 Supplementary methods

1.1 Derivations

The goal of TargetClone is to infer the most likely tree T given AF and SNV measurements:

$$\arg\max_{T} \mathbb{P}(T|\vec{AF}, \vec{SNV}) \tag{1}$$

We infer the best $\vec{C}$ and μ by maximizing:

$$\arg\max_{\vec{C},\mu} \mathbb{P}(\vec{C}, \mu|\vec{LAF}, \hat{T}) \tag{2}$$

Applying Bayes' rule, we can write:

$$\mathbb{P}(\vec{C}, \mu|\vec{LAF}, \hat{T}) = \frac{\mathbb{P}(\vec{LAF}|\vec{C}, \mu, \hat{T})\mathbb{P}(\vec{C}, \mu|\hat{T})}{\mathbb{P}(\vec{LAF}|\hat{T})} \tag{3}$$

$\vec{C}$ and μ are independent, so:

$$\mathbb{P}(\vec{C}, \mu|\vec{LAF}, \hat{T}) = \frac{\mathbb{P}(\vec{LAF}|\vec{C}, \mu, \hat{T})\mathbb{P}(\vec{C}|\hat{T})\mathbb{P}(\mu|\hat{T})}{\mathbb{P}(\vec{LAF}|\hat{T})} \tag{4}$$

μ does not depend on $\hat{T}$:

$$\mathbb{P}(\vec{C}, \mu | L\vec{A}F, \hat{T}) = \frac{\mathbb{P}(L\vec{A}F|\vec{C}, \mu, \hat{T})\mathbb{P}(\vec{C}|\hat{T})\mathbb{P}(\mu)}{\mathbb{P}(L\vec{A}F|\hat{T})} \tag{5}$$

As initially every μ and topology of $\hat{T}$ is equally likely, we do not need to compute the probability of observing μ:

$$\mathbb{P}(\vec{C}, \mu | L\vec{A}F, \hat{T}) \propto \frac{\mathbb{P}(L\vec{A}F|\vec{C}, \mu, \hat{T})\mathbb{P}(\vec{C}|\hat{T})}{\mathbb{P}(L\vec{A}F|\hat{T})} \tag{6}$$

Finally, the probability of $\mathbb{P}(L\vec{A}F|\hat{T})$ is constant with respect to $\vec{C}$ and μ, and we can thus omit the denominator:

$$\mathbb{P}(\vec{C}, \mu | L\vec{A}F, \hat{T}) \propto \mathbb{P}(L\vec{A}F|\vec{C}, \mu, \hat{T})\mathbb{P}(\vec{C}|\hat{T}) \tag{7}$$

1.2 Computing LAF

The LAF measured at position i in subclone j is computed for every element in Q as:

$$LAF_{i,j} = \frac{\min(((1-\mu)L_{Ahi} + \mu L_{Ati}), ((1-\mu)L_{Bhi} + \mu L_{Bti}))}{((1-\mu)L_{Ahi} + \mu L_{Ati}) + ((1-\mu)L_{Bhi} + \mu L_{B}ti)} \tag{8}$$

where L_{Ahi}, L_{Bhi} and L_{Ati}, L_{Bti} are the total number of 'A' and 'B' alleles at position i in the healthy cell and the tumor subclone, respectively.

As the total number of 'A' and 'B' alleles are always equal to 1 in healthy cells, Eq 8 simplifies to:

$$LAF_{i,j} = \frac{\min(((1-\mu) + \mu L_{Ati}), ((1-\mu) + \mu L_{Bti}))}{((1-\mu) + \mu L_{Ati}) + ((1-\mu) + \mu L_{B}ti)} \tag{9}$$

1.3 Gaussian Mixture Model

As our model is based on AF measurements, noise is introduced by variation in the read counts. For WGS data, it has been shown that the read depth can be accurately modeled using binomial and Poission distributions [1, 2]. As was shown by Hajirasouliha et al [3], the read depth can be approximated with a Gaussian distribution when the depth is larger than 1000x, which is typically the case for targeted sequencing data. Therefore, $\mathbb{P}(LAF_{i,j}|C_{i,j}, \mu, \hat{T})$ is modeled as a Gaussian mixture model:

$$\mathbb{P}(LAF_{i,j}|C_{i,j}, \mu, \hat{T}) = \sum_{n=1}^{N} \mathbb{P}(q_{i,j}^{n})\mathcal{N}(\mu_n, \sigma) \tag{10}$$

where N is the total number of possible alleles that can result from $C_{i,j}$, $\mathbb{P}(q_{i,j}^{n})$ is computed using Eq 4 described in the main text. The means of the component μ_n are equal to the LAFs that can be generated from $C_{i,j}$. The noise, σ, is estimated from the LAF measurements in the normal samples of our real TGCC dataset. The interval of the distribution is limited between 0 and 0.5 to adequately model LAF measurements.

1.4 Visualizing $\hat{T}$

For every $\hat{T}$ inferred at every iteration of the algorithm, we sum the event distance between all subclones to obtain a total score. The inferred trees are divided into two groups based on if the ISA could be resolved or not. All trees are sorted within these groups based on the total score. To reduce the amount of information in the final output, only the top 10 trees are reported, where the trees with a resolved ISA are prioritized. The annotated events include gained and lost somatic SNVs, and gained LOH. LOH events are grouped per chromosome arm for clarity. The final visualized output is generated using the Bokeh plotting library.

1.5 Generating TGCC-based simulation data

In addition to a generic simulation dataset, we also generated a simulation dataset based on the expected development of TGCC (see S4B Fig). Starting from a diploid cell, the genome is doubled to form a tetraploid precursor. This precursor further develops into a malignant subclone by acquiring 10 whole chromosome losses, 10 chromosome arm losses and 20 somatic SNVs in the stated order. The affected chromosomes and chromosome arms are randomly selected without replacement. The somatic SNV positions are selected from 36 predefined genomic locations defined based on real observations in our TGCC sequencing dataset. Finally, the malignant subclone acquires 6 copies of chromosome 12p, which is a hallmark of TGCC, and is not allowed to be lost in subsequent cell divisions. All chromosome (arm) losses, gains and somatic SNVs affect only one allele.

In the next step, the malignant precursor continues to divide and form a new subclone. Every subclone also has the capability to divide and form a child subclone for a total of 4 rounds. In each child 8 chromosome arms are gained, 3 chromosome arms are lost, and 2 somatic SNVs are introduced, in this order. If a loss affects an allele containing a somatic SNV, the somatic SNV is lost as well. A subclone is considered unviable if the last allele of a chromosome is lost. In these situations, the subclone is unable to divide further and is removed from the simulation. This process yields 8 subclones (including the healthy cell and precursors) on average.

All generated subclones and precursors are sampled. Every sample is assigned the same predefined tumor fraction. We tested tumor fractions between 0 and 1 in steps of 0.01. A tumor fraction of 0 was included to show the performance of the method on cases where the samples contain no tumor fraction, which may for example occur when it is unclear during sampling if a region contains tumor components or not. Similarly, a tumor fraction of 1 may not be realistic, but provides a reference for how well the method would perform in a perfect world scenario. In each sample, we generate AF and LAF measurements based on the CNV profiles and introduced somatic SNVs. The AF/LAF measurement positions are selected from the real TGCC data.

1.6 Computing the error on the simulation data

The error of $\hat{\vec{C}}$ compared to the true $\vec{C}$ across n measurement positions in m samples is computed as:

$$E_C = \frac{1}{nm} \sum_{i=1}^{n} \sum_{j=1}^{m} |C_{i,j} - \hat{C_{i,j}}| \tag{11}$$

To compute the error in the predicted alleles $\hat{\vec{A}}$, we make use of the event distance as explained in Section "Computing $\mathbb{P}(L\vec{A}F|\vec{C},,\hat{T})$" in the main text:

$$E_A = \frac{1}{nm}\sum_{i=1}^{n}\sum_{j=1}^{m} ED(A_{i,j}, \hat{A_{i,j}}) \tag{12}$$

Similarly, the error of $\hat{\mu}$ for m samples is computed as:

$$E_\mu = \frac{1}{m}\sum_{j=1}^{m} |\mu_j - \hat{\mu_j}| \tag{13}$$

The error of $\hat{T}$ is computed as the ratio of false positive and false negative sample pairs, as described in the main text.

2 Supplementary results

2.1 Combining alleles and somatic SNVs improves performance

To test if the way in which TargetClone combines somatic SNVs and allele information improves performance, we compared our results to results obtained with distance matrices computed from other data types (S7 Fig). In these tests, the MSA was inferred in one method iteration, without attempting to resolve the ISA. For copy numbers, the distance matrix was reconstructed by computing the absolute distance between the inferred copy numbers, incorporating the horizontal dependency, which is detailed in MEDICC. For the alleles, we computed the event distance using the FST shown in Fig 3E. For somatic SNVs, the distance between samples was set to 1 if a relation is possible, or a penalty was assigned if a relation is unlikely as is detailed in Section "Reconstructing T" (Fig 2B). Finally, a distance matrix was also generated by computing the Euclidean distance between the LAF measurements.

We observe that trees based on allele information exclusively are slightly better than those inferred from only copy number information. Both perform much better than trees inferred from somatic SNVs only, and yield a further improvement in error rate compared to trees inferred using the Euclidean distance computed directly on the LAF measurements. However, the error obtained by TargetClone is lower than any other method. This result demonstrates that the combination of distance metrics based on alleles and somatic SNVs, including resolving the ISA, improves tree reconstruction accuracy.

2.2 The number and distribution of measured SNPs does not significantly influence performance

To assess the effect of the number of measured SNPs, we ran additional simulations in which the number of SNPs was increased between 100 and 50000.

As TargetClone was not designed to handle such a large number of SNPs, we segmented the run with 50000 SNPs to chromosome arms and assigned 1 SNP to each arm. A μ of 0.9 was selected for each dataset as the error is low at this tumor fraction in the simulation data and thus it is easier to determine the effect of the number of SNPs. We removed all somatic SNVs from the datasets to avoid potential bias from SNVs during tree reconstruction. S8 Fig shows that the number of SNPs does not have a signficant effect on the performance of TargetClone. Furthermore, as the SNPs are distributed randomly in each simulated dataset, we can observe from the small confidence intervals across the increasing number of SNPs that the effect of the distribution of SNPs is small. In addition, we show in Section 2.4 of S1 Text that the results of our generic simulations (with 500 randomly distrubted SNPs) differs minimally from our results obtained with a TGCC-based simulation (450 SNPs at fixed positions). Thus, we conclude that the effect of the number of distribution of SNPs on the performance of TargetClone is minimal.

2.3 The number of measured SNVs does not significantly influence performance

To test if TargetClone performs better with a specific number of measured SNVs, we performed additional simulations in which the number of measured SNVs was varied between 10 and 10000 SNVs. We selected a μ of 0.9 and a noise level of 0.02. We see no significant change in the performance of TargetClone as the number of input SNVs change (S9 Fig), showing that TargetClone is robust to different numbers of somatic SNVs.

2.4 The TGCC-based simulations yield similar results to the generic simulations

In S22 Fig, we show the mean error and 95% confidence intervals for inferring $\vec{C}$, $\vec{A}$, μ and T in our TGCC-based simulation dataset. The grey shared area shows the mean error and 95% confidence interval for a 100 simulation datasets where the LAF measurements and somatic SNV measurements were assigned a random value between 0 and 1. Interestingly, the error rates differ minimally from the error rates obtained in the generic simulation dataset. Furthermore, these results show that TargetClone is capable of accurately inferring tumor evolution despite the initial duplication to a tetraploid precursor, and the assumption of tetraploidy in the initial tree $\hat{T}1$.

2.5 A comparison of TargetClone to existing methods on targeted sequencing data

First, we corrected the read depth of our TGCC samples using the amplicon sequencing correction methods in CNVKit [4]. However, the segmentation immediately shows that even after such corrections, it remains difficult to properly

detect copy numbers from our read depth data (S15 Fig). In addition, the number of measurements is small and there exists a lot of variation between adjacent measurements, complicating finding a good segmentation.

We attempted to use ASCAT to estimate $\vec{C}$ and μ [5]. However, ASCAT failed to output copy numbers for all but 3 of our 42 samples (S16 Fig). We then used THetA2 on the read depth corrected by CNVKit and the SNP AF to generate $\vec{C}$ and μ estimates assuming 1 tumor subclone per sample [6]. THetA failed on sample EC80 of T6107, NS30 and NS48 of T618 and EC22 of T1382. In S17 and S18 Figs we show a comparison of the copy number estimates between THetA and TargetClone for the most notable results. For sample EC70 of T6107, THeTA estimated a copy number of 0 for chromosomes 12, 15, 18, 21 and 22 with a normal contamination percentage of 0% (S17B Fig). However, with LAF measurements > 0.2 at all of these chromosomes, and somatic SNVs present with a VAF > 0.1 at chromosomes 12 and 15, it seems unlikely that the copy number is truly 0 (S17C Fig). A similar situation can be observed for sample TE74 of T3209. Here, THetA inferred a copy number of 2 everywhere but at chromosome 17 (S18B Fig). However, looking at the raw measurements (S18C Fig), we again do not see clear evidence for these results in the data. The copy numbers reported by TargetClone generally match the raw measurements profile more closely. Overall, the copy numbers are more closely distributed around a copy number of 4, which matches the assumption that TGCC develops by first duplicating to a tetraploid genome.

Although comparing μ is difficult as no ground truth is known, we see a few examples where μ is estimated to be 1 by THetA (S16 Fig). For example, for sample EC70 of T6107, if the copy number were truly 0 at chromosome 12 with a μ of 1, the expected AF of the somatic SNVs would be 0, rather than 0.1 (S17C Fig).

The μ estimated by THetA were used as input to CNVKit to obtain estimates of major and minor copy numbers for the tumor component in each sample. We used these major and minor copy numbers together with somatic SNVs to run PhyloWGS and PyClone.

To make the comparison with PhyloWGS as equal as possible to TargetClone, we assumed that each sample contained 1 tumor subclone and set the cellular prevalence for each CNV equal to the sample μ estimated by THetA. All somatic SNVs that are not shared between all samples had to be excluded from analysis with PhyloWGS. We used 4 chains to run PhyloWGS and the default configuration for PyClone. When running LICHeE, the minimum present VAF was set to 0.0001, and the maximum absent VAF to 0.9999.

Samples for which THetA could not infer a $\vec{C}$ and μ (S16 Fig) are not reported in the PhyloWGS and PyClone-based trees.

In S19 Fig, we show the trees inferred by PhyloWGS. In short, these trees do not match our biological knowledge of TGCC development. The reported tree for T3209 is unlikely as it consists of 3 tumor subclones, while at least 5 different histological elements were sampled for this tumor. For T618, the expected development of FCIS from CIS through BCIS is not reflected in the tree. Additionally, many somatic SNVs are present in sample NS75 only, but

the only reported subclone that is unique to NS75 gains only 1 somatic SNV. For T1382 and T6107, PhyloWGS was unable to infer a tree.

S20 Fig shows the results of coupling LICHeE with cellular prevalences estimated by PyClone. Interestingly, PyClone reports only 3, 3, 4 and 4 subclonal clusters for T3209, T6107, T618 and T1382, respectively, which is fewer than expected for T3209 and T6107 given that we sampled more histological elements than the reported number of subclonal clusters for each patient. For all patients, we see that important relations based on LOH are missed. For T1382, samples MET32, MET35, MET30 and MET32 share LOH on chromosome 6q, 11q and 14q, but this relationship is not visible in the tree. For T6107, LICHeE misses expected relations between the CIS/FCIS and EC samples.

In S21 Fig, the results are shown of running LICHeE on VAFs. For T3209, the relation between all samples other than CIS32 and CIS73 based on LOH on chromosomes 4, 14, 15 and 22 is not captured. For T618, samples NS30, NS48 and NS75 are predicted by LICHeE to have independent origin, but these samples share LOH on chromosomes 9q and 22. All metastasis samples of T1382 are inferred to have originated independently from a healthy cell, while in reality these samples share at least 13 somatic SNVs.

2.6 A comparison of TargetClone to existing methods on simulated data

In addition to real data, we also tested how TargetClone compares to existing methods on simulated data where a ground truth is known. We ran these comparisons on simulated data based on our TGCC data, which resemble targeted sequencing data more directly than our generic simulations. As was shown in Section "The TGCC-based simulations yield similar results to the generic simulations" of S1 Text, the results we obtained on this dataset minimally differ from the results on the generic simulations, and we thus reason that our TGCC-based simulation data is suitable enough to compare methods on.

Since our simulation data do contain ground truth copy number information, it is possible to compare with methods that rely on copy number information and/or somatic SNVs. As MEDICC, a copy number based method, also uses a FST, it is interesting to see how our model compares to this work. From the existing somatic SNV-based methods listed in S1 Fig, 4 methods (PhyloSub, AncesTree, CITUP and LICHeE) are able to reconstruct trees from SNVs in multiple samples. As all of these methods are based on similar principles related to somatic SNVs and no previous study has compared the performance of all 4 methods, we limited our comparison to LICHeE.

To make the comparison fair, we generated a simulation dataset without sequencing noise and normal cell contamination, as the copy numbers used as input to MEDICC do not contain noise and are also not affected by tumor content. For LICHeE, we considered somatic SNVs with a frequency < 0.001 as absent, and a frequency > 0.999 as germline. TargetClone was run with a precursor ploidy of 2.

In contrast to TargetClone, MEDICC and LICHeE reconstruct trees in which all samples are placed at leaf nodes, and thus comparing the resulting trees directly, e.g. by computing the edit distance, is not informative. To enable a meaningful comparison, we therefore converted each tree to a distance matrix and compared the ranked correlation of the distances with the distance matrix of the ground truth tree. For LICHeE, we computed the pairwise distance between samples as the sum of the edge weights across the shortest path between these samples. MEDICC provides a distance matrix based on the CNVs, which we used directly to obtain pairwise sample distances. For TargetClone, we used the distances obtained from distance matrix A_d (see Fig 2B)).

We argue that, if the distance between two samples in the ground truth tree is small, a small distance should also be observed in an inferred tree if the samples are placed correctly. We first ranked each pairwise combination of samples in the ground truth tree by their allelic distance (from matrix A_d). For each tested tool, the sample pairs were ordered according to this ranking, and the ranked distances were correlated with the ground truth (S23 Fig). We see that the distances inferred by TargetClone correlate with the ground truth better than the trees reported by MEDICC and LICHeE. Noteably, in contrast to the ground truth, LICHeE and MEDICC often report a larger distance between the precursor, Germ Cell Neoplasia In Situ (GCNIS) and pre-GCNIS than between GCNIS and its child subclones, resulting in a negative correlation (see S24 Fig for an example). These results do not imply that MEDICC and LICHeE generally perform badly, but that these methods are less suitable to our specific case.

2.7 Comparing TargetClone to existing whole genome sequencing-based methods

To demonstrate the possible benefits of using TargetClone on targeted sequencing data instead of existing methods on WGS data, we used our ovarian cancer dataset, for which both data types are available. We downloaded the aligned BAM files (hg19) and first merged these per patient and filtered unmapped reads using samtools [7]. The sample names in the read groups were corrected using Picard tools [8]. The resulting BAM files were sorted and indexed using samtools. Notably, the reported average read depth of the samples is 3x. SNPs (no indels) were called using samtools mpileup coupled with bcftools call of samtools. These SNPs were filtered for minimum read depth of 30, maximum read depth of 100 and minimum RMS mapping quality of 20 using varFilter of vcfutils of samtools. We ran CNVKit in WGS mode coupled with THetA as described in Section 2.5 of S1 Text to estimate major/minor copy numbers. THetA could not infer a $\vec{C}$ and μ for samples IV3 and V1.

We used Strelka to call somatic SNVs on autosomes and sex chromosomes [9]. These SNVs were filtered for lowEVS or lowDepth. As the runtime of PhyloWGS increases linearly with the number of somatic SNVs, we initially limited the number of SNVs in the PhyloWGS parser to 5000, which did not

complete within reasonable time. Thus, we further reduced the number of SNVs to 1000. For PyClone and LICHeE we used the same settings as discussed in Section 2.5 of S1 Text.

PhyloWGS was unable to infer a tree. Notably, PyClone identifies 3 subclonal clusters, but running LICHeE on the estimated cellular prevalences results in a tree in which all samples originate from the same precursor subclone (S25 Fig). Running LICHeE on VAFs similarly results in a tree in which all samples directly originate from the germline sample (S26 Fig).

We believe that a main reason why the existing WGS tools do not output expected results is related to the low read depth in our dataset. In conclusion, these results show that it may be beneficial to use targeted sequencing coupled with TargetClone in cases where the read depth of WGS analysis is low.

References

[1] Miller CA, Hampton O, Coarfa C, Milosavljevic A. ReadDepth: A Parallel R Package for Detecting Copy Number Alterations from Short Sequencing Reads. PLoS ONE. 2011;6(1):e16327. doi:10.1371/journal.pone.0016327.

[2] Soylev A, Kockan C, Hormozdiari F, Alkan C. Toolkit for automated and rapid discovery of structural variants. Methods. 2017;129:3–7. doi:10.1016/j.ymeth.2017.05.030.

[3] Hajirasouliha I, Mahmoody A, Raphael BJ. A combinatorial approach for analyzing intra-tumor heterogeneity from high-throughput sequencing data. Bioinformatics (Oxford, England). 2014;30(12):i78–86. doi:10.1093/bioinformatics/btu284.

[4] Talevich E, Shain AH, Botton T, Bastian BC. CNVkit: Genome-Wide Copy Number Detection and Visualization from Targeted DNA Sequencing. PLOS Computational Biology. 2016;12(4):e1004873. doi:10.1371/journal.pcbi.1004873.

[5] Van Loo P, Nordgard SH, Lingjaerde OC, Russnes HG, Rye IH, Sun W, et al. Allele-specific copy number analysis of tumors. Proceedings of the National Academy of Sciences. 2010;107(39):16910–16915. doi:10.1073/pnas.1009843107.

[6] Oesper L, Satas G, Raphael BJ. Quantifying tumor heterogeneity in whole-genome and whole-exome sequencing data. Bioinformatics. 2014;30(24):3532–3540. doi:10.1093/bioinformatics/btu651.

[7] Li H. A statistical framework for SNP calling, mutation discovery, association mapping and population genetical parameter estimation from sequencing data. Bioinformatics. 2011;27(21):2987–2993. doi:10.1093/bioinformatics/btr509.

[8] Picard: http://broadinstitute.github.io/picard/.

[9] Saunders CT, Wong WSW, Swamy S, Becq J, Murray LJ, Cheetham RK. Strelka: accurate somatic small-variant calling from sequenced tumornormal sample pairs. Bioinformatics. 2012;28(14):1811–1817. doi:10.1093/bioinformatics/bts271.
